# Effects of praise from a social robot on task persistence in 18- to 24-month-old children

**DOI:** 10.3389/frobt.2026.1782839

**Published:** 2026-02-27

**Authors:** Mikako Ishibashi, Yuta Shinya, Yuichiro Yoshikawa, Hiroshi Ishiguro, Shoji Itakura

**Affiliations:** 1 Department of Psychology and Humanities, College of Sociology, Edogawa University, Chiba, Japan; 2 Graduate School of Education, The University of Tokyo, Tokyo, Japan; 3 Graduate School of Engineering Science, Osaka University, Osaka, Japan; 4 Department of Systems Innovation, Osaka University, Osaka, Japan; 5 Center for Baby Science, Doshisha University, Kyoto, Japan; 6 Research Organization of Open Innovation and Collaboration, Ritsumeikan University, Osaka, Japan

**Keywords:** CommU robot, persistence, praise effect, look, toddlers (18–24 months)

## Abstract

**Introduction:**

Social robots are increasingly being integrated into children’s daily lives, shaping their social interactions and learning behaviors. However, no study has empirically investigated the effect of robot-administered praise in children younger than 4 years old.

**Method:**

This study focuses on the social robot CommU, a simple, approximately 30 cm tall, child-shaped robot that exerts less social pressure and helps children attend to social cues more easily. We examined whether praise from CommU is associated with task persistence in children aged 18–24 months, in comparison with human praise.

**Result:**

Children showed greater task persistence in the Praise condition than in the No Praise condition, regardless of agent type (CommU vs. Human). In addition, children’s task persistence was positively associated with the amount of time they spent looking at the agent.

**Discussion:**

These findings suggest that praise delivered by a social robot is associated with greater task persistence in children aged 18–24 months. Additionally, the positive association between task persistence and time spent looking at the agent suggests that children’s social attention may contribute to sustained engagement during the task. More broadly, the results point to the possibility that social robots may be relevant to aspects of early childhood engagement, beyond the specific task-persistence behavior examined in this study.

## 1 Introduction

Social robots, defined as robots that can interact socially and communicate with humans and other autonomous physical entities (Fong et al., 2003), are being introduced into children’s daily lives (Fridin, 2014; Westlund et al., 2018; Yu and Roque, 2019; Shiomi, 2024), and their presence in homes and classrooms is expected to increase rapidly (Belpaeme et al., 2018; Johal, 2020; van Straten et al., 2020). The various types of social robots include androids, dolls, and animal-like designs, each capable of eliciting beneficial social behaviors from children (Papadopoulos et al., 2020; Nijssen et al., 2021; Sommer et al., 2021).

Robots are more likely to be perceived themselves as social beings when they respond appropriately to children’s actions and words (Leite et al., 2013), behave like friends (Kanda et al., 2004), or express emotions (Hoffman and Ju, 2014). Kumazaki et al. (2020) suggested that when social robots are introduced to young children, small-sized robots with simple designs are preferred. CommU (Vstone Co., Ltd.) is a relatively simple robot that lacks a human-like appearance but has a child-like form, and is approximately 30 cm tall (Uchida et al., 2020). Considering its simple design, the CommU conveys less information than Android robots and is less likely to create social pressure, allowing users to focus more on social cues (Kumazaki et al., 2018a).

Several studies have demonstrated the effectiveness of CommUs in promoting social communication. For instance, conversations with CommUs have facilitated self-disclosure among users (Uchida et al., 2020). Individuals with autism-spectrum disorder (ASD) felt more comfortable communicating with the CommU than with humans (Kumazaki et al., 2018b), suggesting that the CommU may be useful in fostering relationships and providing psychological support. CommU has been applied in learning, where training via the CommU improved joint attention (JA) in children, leading to enhanced performance in subsequent JA tasks with humans (Kumazaki et al., 2019). Thus, the CommU has proven to be an effective tool for supporting children’s learning.

One specific type of support that social robots can provide for children’s social learning is praise. Shiomi et al. (2021) examined the effects of praise received from two robots on children’s learning. Praise is a fundamental social reward that motivates young children to achieve their goals and fosters their desire to learn (Leonard et al., 2021). Shiomi et al. (2021) compared the duration of English learning sessions among children aged 4–6 years under two conditions: one versus two robots praising the children. When two robots praised the children, they spent significantly more time learning English than when only one robot praised them, suggesting that praise from robots encourages persistent learning among young children.

Similarly, other studies have examined the effects of robot-administered praise (Akalin et al., 2019; Davison et al., 2021; Fasola and Matarić, 2010). In these studies, praise was delivered by physically embodied social robots that interacted with users through speech and simple social behaviors such as gaze and gesture. The robots provided encouraging or positive feedback during task performance, often emphasizing effort, engagement, or successful task completion in order to enhance motivation and learning-related outcomes. However, all these studies focused on school-aged or older children (or adults), and little is known about how robot-delivered praise functions in children under 4 years old. Collecting sufficient research data on robots interacting with children under 4 years old is challenging, because of their strong resistance to robots (Kumazaki et al., 2018b). Consequently, with the exception of Tanaka et al. (2007), few studies have explored the interactions between robots and developing children aged 1 or 2 years. Tanaka et al. (2007) placed a robot in a nursery room with children aged 18–24 months over a five-month period. The robot (QRIO) was relatively small (58 cm tall) and its facial structure was similar to that of the CommU, with cameras mounted above both eyes. The robot which was programmed to provide contingent responses to the children over 45 sessions, improved child–robot interaction quality over time, including caretaking behavior and conversation. These findings suggest that even children as young as 18–24 months old can establish social interactions with robots. However, to the best of our knowledge, no study has empirically investigated the effects of robot-administered praise on toddlers (18–24 months old).

The effect of praise on children aged 1–2 years was further examined from the perspective of task persistence. Lucca et al. (2019) conducted an experiment to investigate the impact of parental encouragement on children aged 18–24 months. The results showed that children who received process-focused praise from their parents spent more time working on tasks than those who did not (Lucca et al., 2019). Radovanovic et al. (2023) also found that the persistence of children (17–31 months old) was maximized when parental praise was timed to coincide with their engagement in the task. While previous work highlighted the importance of temporally contingent praise, the present study examined whether repeated, periodic process-focused praise delivered by a physically embodied social robot could support children’s task persistence even without moment-to-moment contingency. In the present study, the robot delivered brief verbal praise statements at fixed intervals during the task, accompanied by simple social cues such as facing the child and maintaining attentive postures. Extending this line of research, we examined whether this type of process-oriented praise from a social robot would be associated with greater persistence in young children, as has been observed in interactions with human caregivers.

With regard to early expressions of persistence, children are widely influenced by interactions with the social environment (Deater-Deckard et al., 2006; Mokrova et al., 2013). Recent research has shown that infants’ behavioral and attentional persistence may be enhanced when they observe persistence in others (Leonard et al., 2017; Shinya and Ishibashi, 2022). Therefore, the effect of praise on children’s persistence may depend on their awareness of others as social beings. For example, Okumura et al. (2023) found that five-year-olds observed by a socially interactive robot shared more stickers than those interacting with an attentional but non-interactive or stationary robot, suggesting that five-year-olds adjust their behavior based on whether the other entity is a social being. However, whether the social awareness of a robot’s presence enhances young children’s persistence remains unclear. In the present study, persistence refers specifically to young children’s continued behavioral engagement with a task in the face of difficulty or lack of immediate success, operationalized as the duration and frequency of their attempts to interact with the task materials.

In this study, we experimentally investigated whether praise from the social robot CommU would be associated with greater persistence in young children aged 18–24 months in the same manner as human praise does. Considering the suggestion that young children prefer small-sized robots (Kumazaki et al., 2020), we found CommU appropriate for children aged 18–24 months. Kumazaki et al. (2018b) pointed out that CommU has a high degree of eye-movement flexibility, indicating that it can easily capture the attention of children. Given that parental encouragement has been shown to enhance task persistence in children aged 18–24 months (Lucca et al., 2019) and that young children perceive robots as social beings (Tanaka et al., 2007), we hypothesized that praise from CommU would be associated with children’s task persistence in a manner similar to human praise. Furthermore, we investigated whether children’s looking behavior toward CommU would be associated with task persistence, based on the expectation that attention to a social agent may be related to children’s engagement and behavioral adjustment (Okumura et al., 2023).

## 2 Methods

### 2.1 Participants

Following a previous study (Lucca et al., 2019), we recruited children aged 18–24 months to participate in this study. In total, 80 children were initially recruited and scheduled for participation. Of these 80 children, 16 were excluded prior to or during the experimental session for the following reasons: (1) fear of the CommU robot that prevented task engagement (*n* = 2); (2) lack of motivation or persistent clinging to their mother for more than 2 min (*n* = 7); and (3) experimental or procedural errors (*n* = 7). Based on an *a priori* sample size estimation, the target sample size was 32 participants per condition. Recruitment was therefore terminated once this target was reached.

Subsequently, during video coding, an additional 8 children were excluded because they did not engage in the entire persistence task and thus did not provide analyzable data. As a result, the final sample used for analysis consisted of 58 children (30 girls; mean age = 20.79 months, *SD* = 2.05). Of these, 30 children were assigned to the CommU condition (mean age = 20.87 months, *SD* = 2.36), and 28 children were assigned to the Human condition (mean age = 20.71 months, *SD* = 1.70). A summary of participant recruitment, exclusions, and final sample characteristics is provided in Table1.

**TABLE 1:** Participant flow and demographic characteristics.

Characteristic	Total sample	CommU condition	Human condition
Initially recruited	80	—	—
Excluded before/During session	16 (2 fear, 7 low motivation/Clinging, 7 procedural errors)	—	—
Target sample size per condition	64	32	32
Excluded during coding	8	—	—
Final analyzed sample (n)	58	30	28
Girls (n)	30	—	—
Mean age (months) (SD)	20.79 (2.05)	20.87 (2.36)	20.71 (1.70)

Based on an *a priori* sample size estimation, the target sample size was 32 participants per condition, and recruitment was terminated once this number was reached. During video coding, an additional eight children were excluded.

This study was approved by the Ethics Committee of Doshisha University (number: 20029).

### 2.2 Stimuli

**FIGURE 1:**
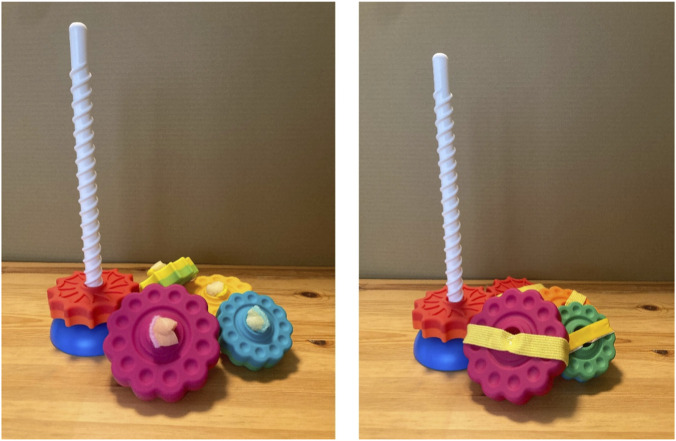
Stacking toy used in the task. Disks were modified with either a sponge (left) or a rubber band (right) to prevent successful stacking.

A toy with stacked gears employed in a previous study (Lucca et al., 2019) was used as a stimulus. This toy consisted of a bar with a base and six disks that could be stacked on top of the bar. To prevent stacking, either a sponge was glued to the center of the disk or a rubber band was glued to the disk (Figure 1).

### 2.3 Procedure

#### 2.3.1 Experimental setup

In the CommU condition, the child, mother, experimental assistant, and CommU were in an experimental room. Before conducting the task, the children were allowed sufficient time (approximately 10–20 min) to interact with CommU to relax. None of the children who participated in the experiment had previously interacted with CommU. The experimental assistant provided support to facilitate the interaction among CommU, mother, and child. After confirming that the child was familiar with CommU (e.g., talking to, laughter, or touching CommU), the experimental assistant prepared the tools used for the persistent task. Under the Human condition, the mother and child were escorted into the experimental room by an experimental assistant.

### 2.4 Persistent task

Two conditions were set, whereby the agent either praised or did not praise the child. The with/without praise condition was a within-participant design, and the order of the conditions was randomized between participants. The agent, CommU/Human, was between participant designs.

#### 2.4.1 Task demonstration

In a typical task, the experimenter first said, “x-chan [child’s name], look” to draw the child’s attention, and then demonstrated how to remove the sponge from the disk and insert the disk into the bar. The experimenter then handed another disk to the child and said, “Here you go, [the child’s name].” After the child received the disk, the child’s behavior was observed for 2 min. To play with the toy, the child needed to insert the disk into the center of the stick; however, a disk glued to sponges or rubber bands cannot be inserted. The sponge and rubber bands were presented in random order. This task was designed to assess task persistence, defined in this study as the child’s continued engagement with a goal-directed activity despite encountering difficulty or lack of immediate success. Because the disks could not be successfully inserted due to the attached sponge or rubber bands, children experienced repeated obstacles during the activity. The 2-min observation period, consistent with the procedure used by Lucca et al. (2019), allowed us to measure how long children remained engaged in the task under these challenging conditions, which was operationalized through their trying behavior.

### 2.5 CommU condition

#### 2.5.1 Praise condition

The CommU praised the child every 10 s after the disk was handed to him/her. The timing of the praise was fixed at every 10 s for 2 min. Praise was given in the following order: “[child’s name], you are doing well”; “[child’s name], keep it up”; and “[child’s name], you’re working hard.” These utterances constituted process-focused praise, as they emphasized the child’s ongoing effort and engagement rather than success, ability, or task completion. The phrases were selected so that they would remain appropriate regardless of whether the child was able to successfully complete the task. In a study by Shiomi et al. (2021), a human operator was incorporated into the system to control the timing of the robot’s actions because children behaved unexpectedly. As in the study by Shiomi et al. (2021), the timing of the praise was fixed (once every 10 s). We selected sentences that were not unnatural even when the child was attempting to perform an unattainable task. The operator remotely controlled the CommU and moved the CommU’s face and body appropriately in response to the child’s movements, allowing the praise to be presented as part of a socially responsive interaction. After 2 min, the experimenter again drew the child’s attention and handed the child a disk that could be inserted into the stick to reduce frustration.

#### 2.5.2 No Praise condition

The disks and stick with base used in the Praise condition were removed, and new disks and stick with base were placed on the floor. The disks had a rubber band attached to them, and the experimenter demonstrated the removal of the rubber band attached to the disk. Measurements began after the child was provided with a disk. Similar to the Praise condition, the face and body moved in accordance with the child’s movements.

### 2.6 Human condition

#### 2.6.1 Praise condition

A human praised the child every 10 s after handling the disk. The lines and order of praise were the same as in the CommU condition. After 2 min, the experimenter again drew the child’s attention and handed the child a disk that could be inserted into the stick to reduce frustration.

#### 2.6.2 No Praise condition

The disks and stick used in the Praise condition were removed and new disks and stick with base were placed. The disks had a rubber band attached to them, and the experimenter demonstrated the removal of the rubber band attached to the disks. Measurements began after the disks were handed over to the child. As in the Praise condition, the face and body moved in accordance with the child’s movements.

### 2.7 Coding schema

#### 2.7.1 Trying

Children’s persistence was operationalized as “Trying,” was measured as described by Lucca et al. (2019) for the following two behaviors:

1. Inserting disk into the stick: Trying was coded when the disk was placed at the tip of the stick. The start time was when the child placed the disk at the tip of the stick, and the end time was when the disk left the tip of the stick.
2. Removal of the sponge/rubber band from the disks: The time at which each child attempted to remove the sponge or rubber band glued to a disk was recorded. The start time was when the child tried putting a finger on the sponge or removed the rubber band, and the end time was when the child’s finger left the disk.

#### 2.7.2 Look

We measured the duration of the children’s looking time in the CommU/Human condition. Look commenced when the child looked at the CommU (CommU condition) or a human (Human condition) for at least 1 s.

## 3 Results

**FIGURE 2:**
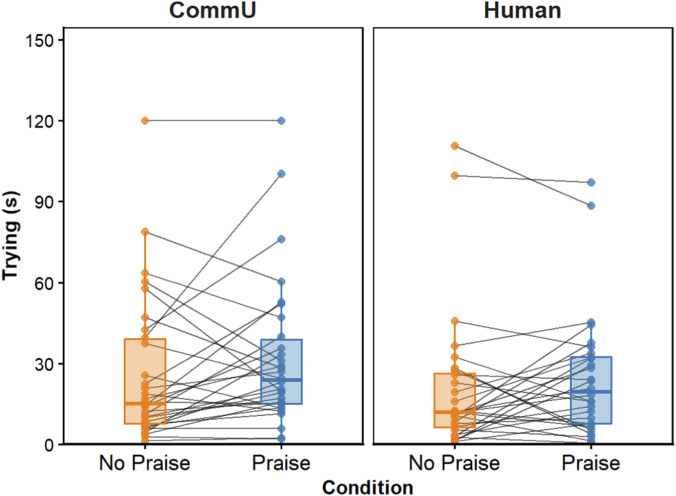
Mean time (s) of Trying for the No Praise and Praise condition per Agent.

Descriptive statistics are shown for each Agent with Praise and No praise Condition (Table 2), along with the mean time spent Trying between the No Praise and Praise conditions for each Agent (Figure 2).

**TABLE 2:** Mean and standard deviation of Trying and Look with Praise and No Praise condition per Agent.

Agent	Condition	Mean of Trying (*SD*)	Mean of Look (*SD*)
CommU	No praise	26.3 (27.31)	12.01 (11.97)
CommU	Praise	32.22 (27.48)	11.86 (10.1)
Human	No praise	21.04 (26.42)	7.84 (7.35)
Human	Praise	24.91 (23.28)	8.00 (8.7)

**FIGURE 3:**
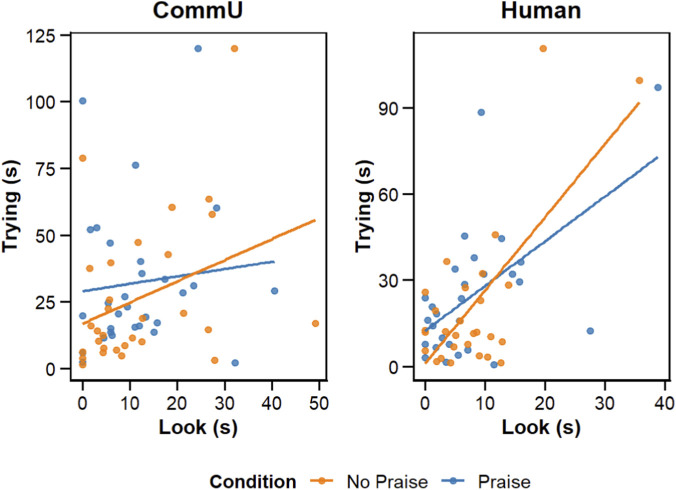
Pearson’s correlation between Trying and Look in the CommU (left) and Human (right) conditions. Orange = No Praise condition, Blue = Praise condition.

We measured Look during the task and examined the relationship between the length of Look and Trying using Pearson’s correlation analysis. In the CommU condition, Pearson’s correlation analysis revealed no significant correlation between Trying and Look during the Praise (*r* = 0.10, *p* = 0.59) and No Praise (*r* = 0.35, *p* = 0.06) conditions. In the Human condition, the correlation analysis revealed a significant correlation between Trying and Look in the Praise (*r* = 0.58, *p* = 0.001) and No Praise (*r* = 0.71, *p* = 0.001) conditions (Figure 3).

### 3.1 Analysis

Linear mixed-effects models (LMMs) were employed using the lmer function from the lme4 package in R (Bates et al., 2015) to examine the effects of age, sex, Condition (Praise/No Praise), Look, and Agent (CommU/Human) on the Trying. In these models, fixed effects represent average effects of the predictors across participants, that is, the general tendencies observed after accounting for individual differences, while random intercepts for participants account for individual differences in baseline Trying. Continuous predictors (Age and Look) were standardized (z-scored) prior to analysis. For each fixed effect, *B* represents the estimated regression coefficient, indicating the expected change in the outcome associated with a one-unit increase in the predictor, or the difference from the reference category for categorical predictors. *SE* denotes the standard error of the coefficient. The *t* value represents a Wald statistic calculated as the ratio of the coefficient to its standard error (*B/SE*), which reflects the strength of evidence for the effect.

We included the participants as random intercepts to account for individual differences. Age and Look variables were standardized using z-scores. Model 1 was used as a baseline model to assess the effects of standardized Age and Sex on the Trying. A random intercept for participants (ID) was included as an individual difference. Model 2 was an extension of Model 1 with condition (Praise/No Praise), Standardized Look, and Agent (CommU/Human), added as fixed effects. Model 3 further explained the interaction to examine the effects of the Condition and Look on the agent’s trying behavior. Each model is described in detail in the Appendix.

Table 3 presents the LMM results. In Model 1, standardized Age is positively associated with Trying (*B* = 8.06, *SE* = 3.11, *t* = 2.59, *p* = 0.01), indicating that older children tended to engage in more trying behavior. Sex does not show a significant effect (*B* = −7.18, *SE* = 6.22, *t* = −1.16, *p* = 0.25).

**TABLE 3:** Comparison of LMMs.

Variables	Model 1			Model 2			Model 3		
*Predictors*	*B*	95%CI	*P*	*B*	95%CI	*P*	*B*	95%CI	*P*
(Intercept)	29.78	21.24–38.31	**<0.001**	26.08	16.45–35.72	**<0.001**	26.01	16.18–35.84	**<0.001**
Standardized Age	8.06	1.90–14.22	**0.011**	7.15	1.54–12.76	**0.013**	6.58	0.99–12.16	**0.021**
Sex [m]	−7.18	−19.46–5.10	0.249	−4.17	−15.45–7.10	0.465	−3.59	−14.74–7.57	0.525
Condition [Praise]				5.09	0.23–9.95	**0.040**	6.24	−0.57–13.04	0.072
Standardized Look				10.61	4.80–16.41	**<0.001**	7.29	0.18–14.41	**0.045**
Agent [human]				−0.63	−12.10–10.85	0.914	1.16	−11.21–13.52	0.853
Condition [Praise] x Agent [human]							−2.37	−12.17–7.42	0.632
Agent [human] x standardized Look							9.34	−2.57–21.26	0.123
Observations	116			116			116		
Marginal R^2^/Conditional R^2^	0.108/0.746			0.264/0.764			0.287/0.764		
AIC	1046			1034			1035		

The Bold font indicates statistical significance (p < 0.05).

In Model 2, standardized Age remains significant (*B* = 7.15, *SE* = 2.83, *t* = 2.53, *p* = 0.01). In addition, Condition (Praise vs. No Praise) and Look are significant predictors of Trying (Condition: *B* = 5.09, *SE* = 2.45, *t* = 2.08, *p* = 0.04; Look: *B* = 10.61, *SE* = 2.93, *t* = 3.62, *p* = 0.001), indicating that children in the Praise condition and those who looked at the agent for longer tended to engage in more trying behavior.

In Model 3, standardized Age, Condition, and Look remain significant predictors of Trying (Standardized Age: *B* = 6.58, *SE* = 2.82, *t* = 2.34, *p* = 0.02; Look: *B* = 7.29, *SE* = 3.59, *t* = 2.03, *p* = 0.05). The effect of the condition showed a marginal trend (*B* = 6.24, *SE* = 3.43, *t* = 1.82, *p* = 0.07). No significant interaction effect is observed between Agent and Condition (*p* = 0.63) or between Agent and Look (*p* = 0.13), indicating that the effects of Praise and Look did not differ between the CommU and Human. The main effect of Condition, which is significant in Model 2 becomes a meaningful trend (*p* = 0.07).

Model comparisons were conducted using likelihood ratio tests (LRTs), which evaluate whether a more complex model provides a significantly better fit to the data than a simpler, nested model. In the LRTs, Model 2 exhibits a significantly improved fit compared to Model 1 (χ^2^ (3) = 17.79, *p* < 0.001). However, no significant effect is observed between Model 2 and Model 3 (χ^2^ (2) = 2.87, *p* = 0.24), suggesting that the effect of interaction of Model 3 does not improve significantly. Model fit was also evaluated using the Akaike Information Criterion (AIC), a measure that balances model fit and model complexity, with lower values indicating better fit. Model 2 showed the lowest AIC (*AIC* = 1034), suggesting that it provided the best overall balance between explanatory power and parsimony.

## 4 Discussion

This study aimed to examine whether children aged 18–24 months show greater task persistence in task-related behavior when praised by the social robot CommU, similar to when praised by a human. We examined the effects of Condition (Praise/No Praise), Agent (CommU/Human), and Look (children’s looking time at the agent), as predictors of children’s persistence in their tasks. Using the LMM, we found that Age, Condition (Praise/No Praise), and children’s Look toward the agent were significantly associated with task persistence. Specifically, persistence tended to increase with age, and children showed greater persistence when they received praise and when they looked at the Agent for longer periods. Importantly, model comparison based on the AIC indicated that a model including only these main effects (Model 2) provided the best balance between explanatory power and parsimony. Accordingly, adding interaction terms between Agent and Condition or between Look and Condition did not improve model fit, suggesting that the associations between Praise, Look, and task persistence were similar across Agent types and conditions.

Our results suggest that the praise of CommU is associated with greater task persistence in children aged 18–24 months, and is consistent with previous research (Lucca et al., 2019), which found that young children who received parental praise persisted in their tasks for a longer period. This also aligns with Shiomi et al. (2021), who demonstrated that children aged 5–6 years spend more time working on a task when encouraged by social robots. Interestingly, our study found that praise from CommU was associated with greater task persistence compared to the No Praise condition. This suggests that even children as young as 18–24 months of age may respond to social signals from CommU.

In addition, regardless of whether CommU provided praise, the children were drawn to the robot, which led them to look at it. Okumura et al. (2023) found that five-year-olds engaged in strategic reputation management by sharing more stickers when observed by a social robot than by a non-interactive robot. This suggests that the presence of a robot is relevant to children’s attention to social cues and may be related to their social behavior.

Similarly, Kumazaki et al. (2018b) noted that CommU’s high degree of eye-movement freedom naturally draws user attention. Anzalone et al. (2014) used the Nao robot, a design relatively similar to CommU, to train children with ASD and found that the JA scores in children with ASD significantly decreased when using Nao. Since Nao’s eyes are smaller than CommU’s, children with ASD may focus on the non-eye-related features of Nao (Pennisi et al., 2016). Considering these studies, CommU’s eye features may have increased children’s social awareness, contributing to their engagement in the task.

A significant Age effect was also observed, consistent with the findings of Radovanovic et al. (2023), who demonstrated a linear relationship between age and engagement in a task among children aged 17–31 months. Previous studies have suggested that as children aged 1–2 years grow, they develop greater effortful control (Rothbart et al., 2000; Rothbart and Bates, 2007; Putnam et al., 2024). Hence, task persistence can be assumed to be related to the age of children in this age group.

Previous studies have suggested that young children prefer small and simply designed robots (Kumazaki et al., 2020). Additionally, children aged 1–2 years with fewer preconceptions about robots may engage in more fundamental social interactions that do not rely on advanced conversation (Tanaka et al., 2007). Tanaka et al. (2007) used a small, simple-faced robot (QRIO) to investigate the qualitative changes over 4 months in interactions with children aged 18–24 months. They found that robots with simpler appearances allow children to focus more on social cues, because processing excessive visual information can be challenging at this age (Kumazaki et al., 2020). Similarly, in the present study, the CommU’s simple design may have allowed children to focus on social cues such as Praise and looking behaviors.

Communication with robots, which is less complex than that with humans, is processed at an “intermediate difficulty” level, making robot interactions more accessible to young children (Dubois-Sage et al., 2024). To further investigate these considerations, future research should examine the persistence of children aged 18–24 months using robots other than the CommU.

One limitation of this study is that we could not conclusively determine whether the social reward of praise from CommU directly increased children’s persistence. Lucca et al. (2019) argue that process-oriented praise teaches children the importance of their efforts. However, whether the effect observed in our study was due to process-oriented vocalizations or simply the presence of vocal praise remains unclear. Future research should include control conditions in which CommU provides meaningless utterances during tasks, to isolate the effects of praise as a social reward.

In addition, as in previous studies, predicting young children’s responses was difficult; therefore, we did not implement contingent responses (Shiomi et al., 2021). In this study, praise was not provided contingently to avoid cases where the children assigned to the Praise condition did not engage with the disk and therefore would not receive praise. Prior research suggests that praise timing matters, and praise given during a behavior is more effective in increasing persistence than praise given afterward (Radovanovic et al., 2023). Future studies can compare a randomly moving robot with a robot that provides contingent praise based on children’s responses.

Moreover, in this study, the experiments began only after confirming that the children had become familiar with CommU (e.g., a child-speaking CommU). However, previous research suggests that young children (aged 1–2 years) may require more time to develop emotional bonds (Ahmad et al., 2019), a sense of closeness (Kose-Bagci et al., 2009), and psychological attribution (Nijssen et al., 2021; Okumura et al., 2023). Although few studies have explored the individual differences in children’s interactions with robots, Baxter et al. (2017) highlighted the importance of these factors. Tolksdorf et al. (2021) found that shy children initially exhibit fewer positive reactions to robots than non-shy children, but become more comfortable over time. Therefore, future research should consider individual differences such as personality, temperament, and familiarity with robots, when examining task persistence.

## 5 Conclusion

This study offers initial evidence for a potential link between praise from CommU and task persistence in children aged 18–24 months. We also found, for the first time, that longer looking toward the Agent was associated with greater task persistence in children, regardless of Agent type. Although caution is required when interpreting whether praise from a robot serves as a social reward equivalent to human praise (Dautenhahn, 2007), our findings suggest that CommU, as a simplified social agent, may be relevant to children’s sensitivity to social signals at 1–2 years of age. The results of this study point to the possibility that social robots may play a role in supporting aspects of early childhood engagement beyond the specific task-persistence behavior examined here. Future research should examine how the timing and contingency of robotic praise influence children’s persistence and explore how social robots may contribute to early childhood play and learning in broader contexts.

## Data Availability

The raw data supporting the conclusions of this article will be made available by the authors, without undue reservation.
