# Supplementary material for: Effects of praise from a social robot on task persistence in 18- to 24-month-old children
Source: Front Robot AI. 2026 Feb 27;13:1782839. doi: 10.3389/frobt.2026.1782839 (PMC12982925; doi:10.3389/frobt.2026.1782839)
Supplement: Supplementary file 1 [file Supplementaryfile1.pdf]

Supplementary file

Specification of Linear Mixed-Effects Models

- The linear mixed-effects models compared in the main analyses are described below. All models were fitted using the lmer function from the lme4 package in R.

Model 1 (Baseline model)

$$\text{Trying}_{ij} = \beta_0 + \beta_1(\text{Standardized Age}_{ij}) + \beta_2(\text{Sex}_{ij}) + u_{0j} + \varepsilon_{ij}$$

- Model 1 served as a baseline model to assess the effects of standardized age and sex on trying behavior. A random intercept was included for participants (ID) to account for individual differences in baseline trying behavior.

Model 2 (Main-effects model)

$$\text{Trying}_{ij} = \beta_0 + \beta_1(\text{Standardized Age}_{ij}) + \beta_2(\text{Sex}_{ij}) + \beta_3(\text{Condition}_{ij}) + \beta_4(\text{Standardized Look}_{ij}) + \beta_5(\text{Agent}_{ij}) + u_{0j} + \varepsilon_{ij}$$

- Model 2 extended Model 1 by adding Condition (Praise vs. No Praise), Standardized Look, and Agent (CommU vs. Human) as fixed effects.

Model 3 (Interaction model)

$$\text{Trying}_{ij} = \beta_0 + \beta_1(\text{Standardized Age}_{ij}) + \beta_2(\text{Sex}_{ij}) + \beta_3(\text{Condition}_{ij} \times \text{Agent}_{ij}) + \beta_4(\text{Standardized Look}_{ij} \times \text{Agent}_{ij}) + u_{0j} + \varepsilon_{ij}$$

- Model 3 further examined whether the effects of Condition and Look on trying behavior differed as a function of the Agent by including interaction terms.

Note.

- The term u_{0j}represents the participant-specific random intercept corresponding to the random effect specified as (1 | ID) in the lmer models. The residual term ε_{ij}represents observation-level error and is implicitly assumed in all models.
- Age and Look were standardized prior to analysis.

- Condition was coded as Praise vs. No Praise. Agent was coded as CommU vs. Human.
